# Don't show us your instrument park: Give us your students/give us to your students!

**DOI:** 10.1111/1751-7915.14326

**Published:** 2023-08-16

**Authors:** Victor de Lorenzo, Kenneth Timmis

**Affiliations:** ^1^ Systems Biology Department Centro Nacional de Biotecnología (CNB‐CSIC) Madrid Spain; ^2^ Institute of Microbiology Technical University of Braunschweig Braunschweig Germany

Inviting colleagues from other institutions to partake in seminars or consortial research meetings holds immense significance in the realms of information sharing, strategic research planning and, perhaps most importantly, in stimulating creativity through brainstorming and exploring the vast expanse of *idea space*. These interactions often serve as catalysts for novel experiments, fresh directions and exciting collaborations. Social events accompanying these visits offer a more relaxed setting and a different context for discussions, which can unexpectedly trigger new and ground‐breaking ideas, and of course can create new/strengthen existing synergistic relationships. It is within the time spent with postgraduate students and postdocs that maximum reciprocal benefits are often reaped, as it provides a chance for budding researchers to learn from and engage with established experts, and for visitors to immerse themselves in the enthusiasm and fresh ideas flourishing among young scientists.

The value of these professional visits with either close or distant colleagues cannot be overstated. However, the pressing challenge lies in the time constraints, as such exchanges are typically limited to a few hours. To ensure these interactions are truly productive and enjoyable, let us explore some experience‐based hints to maximize their potential.

Foremost, it is essential to avoid squandering precious time showcasing the facilities and instruments in your laboratory or research centre, regardless of how proud you are of them. While laboratories and equipment vary, dedicating substantial portions of the visit to guided tours of in‐house facilities is wasteful when time is of the essence. Instead, focus on meaningful discussions that delve into research questions, advances and challenges, and the ideas to which visitors can contribute. Unless the visit explicitly centres around discussing specific technologies, spending time admiring infrastructure is time of missed opportunities.

Second, make the most of the time shared with visitors by tapping into their expertise and seeking their opinions and advice on ongoing projects, particularly those undertaken by early career researchers. Valuable ideas frequently emerge during these discussions, proving that brainstorming together among people endowed with diverse skills and expertise is an unbeatable source of innovation. Additionally, take advantage of any remaining time for socializing and building personal connections. Face‐to‐face interactions with colleagues at the forefront of their respective fields hold a unique value that cannot be replaced by AI, Google searches or even reading the most impactful literature. Some of these connections develop into long‐term associations that often become indispensable for navigating the research landscape along one's career.

Third, serendipity undoubtedly plays a significant role in science: the chance meeting of ideas/expertise/technologies at an opportune moment that becomes a *light bulb* occasion. Serendipity cannot be planned but is favoured by researcher interactions. Similarly, *Eureka moments*, those instances when one suddenly realizes the meaning of a collection of data that previously did not make any sense, are equally cherished by all researchers. These non‐anticipated breakthroughs can emerge in the minds of both experienced scientists and their younger counterparts with equal probability during a scientific conversation; not grabbing the opportunity of mutual illumination is a heavy loss for both sides (how often do we hear: ‘*Brilliant! We had similar data but did not consider that possibility*.’?).

Fourth, it is vital to distinguish between audiences when presenting your facilities and discussing your research. While politicians and funders may be captivated by photoshoot opportunities with grand machines, instrument parks rarely excite knowledgeable and intelligent researchers. Instead, they are more likely to appreciate the sharing of new ideas and approaches. In fields like Biology, where progress hinges on talent and creativity, conversations with individuals from diverse backgrounds play a pivotal role in awakening breakthrough concepts. In particular, encourage early career scientists to actively engage in discussions with visitors, promoting a mutual exchange of questions and out‐of‐the‐box ideas. As Linus Pauling aptly put it ‘*… If you want to have good ideas you must have many ideas. Most of them will be wrong, and what you have to learn is which ones to throw away…*’ And visits to other research centres are the privileged scenario for this to happen, for the sake of both the visitor and the visited!

In essence, when planning a visit, prioritize organizing it to maximize intellectual interactions and brainstorming sessions, and leave shows of technological muscle aside. Emphasize research questions, advancements, problems and plans to unlock the full potential of these visits for both parties involved. In this regard, the interactions with students and postdocs becomes an invaluable element, enriching both the visiting and host institutions.

In conclusion, professional visits between research centres should be carefully designed to encourage spending enough quality time together among participants. By shifting the focus from facility tours to substantive discussions and idea sharing, these visits can lead to transformative breakthroughs and enduring partnerships. Embrace the power of face‐to‐face interactions, and let these exchanges serve as a catalyst for intellectual growth and scientific advancement.

*Give us your students/give us to your students!*

## CONFLICT OF INTEREST STATEMENT

The authors have no competing interests.

